# Neoadjuvant modified FOLFIRINOX followed by postoperative gemcitabine in borderline resectable pancreatic adenocarcinoma: a Phase 2 study for clinical and biomarker analysis

**DOI:** 10.1038/s41416-020-0867-x

**Published:** 2020-05-20

**Authors:** Changhoon Yoo, Sang Soo Lee, Ki Byung Song, Jae Ho Jeong, Jaewon Hyung, Do Hyun Park, Tae Jun Song, Dong Wan Seo, Sung Koo Lee, Myung-Hwan Kim, Seung Soo Lee, Jin Hee Kim, Hyung-seung Jin, Jin-hong Park, Dae Wook Hwang, Jae Hoon Lee, Woohyung Lee, Heung-Moon Chang, Kyu-pyo Kim, Baek-Yeol Ryoo, Song Cheol Kim

**Affiliations:** 1 Department of Oncology, Asan Medical Center, University of Ulsan College of Medicine, Seoul, Korea; 2 Department of Gastroenterology, Asan Medical Center, University of Ulsan College of Medicine, Seoul, Korea; 3 Department of Surgery, Asan Medical Center, University of Ulsan College of Medicine, Seoul, Korea; 4 Department of Radiology, Asan Medical Center, University of Ulsan College of Medicine, Seoul, Korea; 5 Department of Convergence Medicine, Asan Institute for Life Sciences, Asan Medical Center, University of Ulsan College of Medicine, Seoul, Korea; 6 Department of Radiation Oncology, Asan Medical Center, University of Ulsan College of Medicine, Seoul, Korea

**Keywords:** Pancreatic cancer, Surgical oncology

## Abstract

**Background:**

Patients with borderline resectable pancreatic cancer (BRPC) have poor prognosis with upfront surgery.

**Methods:**

This was a single-arm Phase 2 trial for clinical and biomarker analysis. The primary endpoint is 1-year progression-free survival (PFS) rate. Patients received 8 cycles of neoadjuvant modified (m) FOLFIRINOX. Up to 6 cycles of gemcitabine were given for patients who underwent surgery. Plasma immune cell subsets were measured for analysing correlations with overall survival (OS).

**Results:**

Between May 2016 and March 2018, 44 chemotherapy- and radiotherapy-naïve patients with BRPC were included. With neoadjuvant mFOLFIRINOX, the objective response rate was 34.1%, and curative-intent surgery was done in 27 (61.4%) patients. With a median follow-up duration of 20.6 months (95% confidence interval [CI], 19.7–21.6 months), the median PFS and OS were 12.2 months (95% CI, 8.9–15.5 months) and 24.7 months (95% CI, 12.6–36.9), respectively. The 1-year PFS rate was 52.3% (95% CI, 37.6–67.0%). Higher CD14^+^ monocyte (quartile 4 vs 1–3) and lower CD69^+^ γδ T cell (γδ TCR^+^/CD69^+^) levels (quartiles 1–3 vs 4) were significantly associated with poor OS (*p* = 0.045 and *p* = 0.043, respectively).

**Conclusions:**

Neoadjuvant mFOLFIRINOX followed by postoperative gemcitabine were feasible and effective in BRPC patients. Monocyte and γδ T cells may have prognostic implications for patients with pancreatic cancer. ClinicalTrials.gov identifier: NCT02749136.

## Background

Pancreatic cancer is the seventh leading cause of cancer-related death worldwide.^1^ The overall 5-year survival rate is less than 6% due to late clinical manifestations and the systemic nature of the disease at presentation. Although surgery is the only curative treatment option for resectable disease, 5-year survival rates after resection remain poor between at 15 and 30%.^2–4^

Borderline resectable pancreatic cancer (BRPC) and locally advanced unresectable pancreatic cancer (LAUPC) are anatomically characterised by the involvement extent of major vessels, which is likely associated with positive resection margin.^5^ BRPC has been regarded as a potentially curative disease, and neoadjuvant therapy may lead to improvements in R0 resection rates and long-term survival. Although there is no evidence based on randomised Phase 3 trials investigating BRPC or LAUPC, conventional or modified FOLFIRINOX has been widely used as neoadjuvant therapy based on its success with metastatic pancreatic cancer patients.^6^ However, the optimal duration of preoperative chemotherapy, regimens of postoperative chemotherapy, and biomarkers for the prediction of prognosis have not yet been defined.

We investigated the efficacy and safety of perioperative chemotherapy consisting of neoadjuvant modified FOLFIRINOX (mFOLFIRINOX) and postoperative gemcitabine for patients with BRPC. Biomarker analyses using peripheral blood immune cell subsets were performed to assess their prognostic implications.

## Methods

### Patients

This was a single-centre, single-arm, Phase 2 trial. Patients with cytologically or histologically proven pancreatic ductal adenocarcinoma were prospectively enrolled if they met the following inclusion criteria: radiographically documented borderline resectable status according to the National Comprehensive Cancer Network (NCCN) criteria^7^ determined by academic gastrointestinal radiologists; Eastern Cooperative Oncology Group performance status (ECOG) 0 or 1; no prior chemotherapy or radiotherapy for pancreatic cancer; and adequate bone marrow, renal, and hepatic function. Patients with adenosquamous carcinoma or neuroendocrine carcinoma and those with evidence of distant metastasis based on computed tomography (CT), magnetic resonance imaging (MRI), or ^18^F-fluoro-2-deoxyglucose (FDG)–positron emission tomography (PET)-CT, were excluded. In this study, baseline ^18^F-FDG-PET-CT was mandatory to exclude the distant metastases based on the findings from the previous report.^8^ This study was approved by the Asan Medical Center Institutional Review Board (approval number: 2016-0010), and all patients provided written informed consent.

### Treatment

#### Neoadjuvant mFOLFIRINOX

A total 8 cycles of mFOLFIRINOX was administered every 14 days prior to surgery. Fluorouracil was administered as a 2400 mg/m^2^ continuous infusion for 46 h. Leucovorin 400 mg/m^2^, oxaliplatin 85 mg/m^2^, and irinotecan 150 mg/m^2^, were administered on day 1. Primary granulocyte-colony stimulating factor (G-CSF) support was not allowed. Dose interruptions and reductions in response to the adverse events (AEs) were predefined in the protocol. Response evaluations using CT or MRI of the abdomen and pelvis were performed every 4 cycles of mFOLFIRINOX.

#### Surgery

After 8 cycles of mFOLFIRINOX, imaging findings (CT, MRI, and FDG-PET-CT) were reviewed for surgical resectability by a multidisciplinary team, including pancreatobiliary surgeons, gastroenterologists, radiologists, medical oncologists, and radiation oncologists. For patients considered to have resectable tumours after the review, surgery was performed 4–6 weeks after the last dose of mFOLFIRINOX. The extent of surgical resection was decided by attending surgeons. Pathologic findings, including margin status and nodal status, were graded by institutional standards, which followed the guidelines of the American Joint Committee on Cancer, 8th edition. R1 resection was defined as the microscopic evidence of tumour within 1 mm of a resection margin.^9^ For patients whose tumours remained unresectable after 8 cycles, mFOLFIRINOX was continued until disease progression or unacceptable toxicity. For these patients, radiotherapy such as concurrent chemoradiotherapy or stereotactic body radiotherapy therapy was also permitted.

#### Postoperative gemcitabine

For patients who underwent surgical resection, 3 cycles of gemcitabine were administered at 1000 mg/m^2^ as a 30-min weekly infusion for 3 consecutive weeks every 4 weeks. At the discretion of the attending physician, up to 6 cycles of gemcitabine were allowed. During postoperative gemcitabine administration, CT scans and serum CA 19-9 measurements were performed every 3 cycles.

#### Radiotherapy

Preoperative radiotherapy was not administered in this study. Postoperative concurrent chemoradiotherapy with intravenous fluorouracil was administered for patients with R1 resection following the completion of planned postoperative gemcitabine.

#### Follow-up

For patients who underwent surgery, CT scans and serum CA 19-9 measurements were performed every 3 months for the first 2 years, then every 6 months for the subsequent 3 years. For patients who did not undergo surgery after preoperative mFOLFIRINOX, CT scans and serum CA 19-9 measurements were conducted every 4 cycles of mFOLFIRINOX for patients treated with mFOLFIRINOX, or every 2 months for patients who discontinued mFOLFIRINOX without disease progression. Additional imaging studies were conducted whenever clinically indicated.

### Biomarker analysis

For biomarker analysis, 15 mL of blood was collected at baseline and at the time of each disease evaluation. Peripheral blood mononuclear cells (PBMCs) were obtained by standard density gradient centrifugation. To evaluate the prognostic implications of immune cells, multicolour flow cytometry analyses using the CytoFLEX flow cytometry platform (Beckman Coulter, Brea, CA) were performed to determine the proportions of different immune cell populations in the PBMCs. Panels for multicolour flow cytometry included CD3 (UCHT1; BioLegend, San Diego, CA, USA), CD4 (OKT4; BioLegend), CD8 (SK1; BioLegend), CD14 (63D3; BioLegend), CD11c (3.9; BioLegend), CD56 (5.1H11; BioLegend), γδ TCR (B1; BioLegend), HLA-DR (L243; BioLegend), CD69 (FN50; BioLegend), FoxP3 (236A-E7; eBioscience, San Diego), PD-1 (EH12.2H7; BioLegend), LAG-3 (11C3C65; BioLegend), CTLA-4 (L3D10; BioLegend), and TIGIT (A15153G; BioLegend).

### Blinded central image review

An independent central imaging review for borderline resectable status was not mandatory for the enrolment. After patient enrolment was completed, blinded baseline imaging data were subsequently collected and reviewed centrally by two academic pancreatobiliary radiologists (S.S.L. and J.H.K.) for disease extent according to the NCCN criteria.^7^

### Statistical analysis

Based on the results of our previous BRPC study,^10^ the 1-year progression-free survival (PFS) rate was estimated to be 30% (P0) with gemcitabine-based neoadjuvant chemotherapy. With neoadjuvant mFOLFIRINOX, the 1-year PFS rate was estimated to improve to 50% (P1). To detect this difference with a 2-sided alpha of 0.05 and a power of 80%, 39 patients were required. Assuming a 10% of drop-out rate, a total of 44 patients were required for this study.

PFS was defined as the time between the start of first cycle of neoadjuvant mFOLFIRINOX and disease progression defined by the Response Evaluation Criteria in Solid Tumors), version 1.1 (RECIST v1.1) or any cause of death, whichever occurred first. Overall survival (OS) was defined as the period from the start of the first cycle of neoadjuvant mFOLFIRINOX until death from any cause. Tumour responses were graded by the treating investigators according to RECIST v1.1.

Analyses of efficacy outcomes were based on the full analysis set (FAS), which consisted of all patients who received at least one dose of study drug. The safety set consisted of all patients who received at least one dose of study drug and had at least one valid post-baseline safety assessment. AEs were graded according to the National Cancer Institute Common Terminology Criteria for Adverse Events (CTCAE), version 4.03. The Kaplan–Meier method was used to estimate PFS and OS, with surviving patients censored at the time of last follow-up. For exploratory analyses, the log-rank test and cox-proportional hazards regression model were used to evaluate the association with survival outcomes. Multivariate analysis was not performed because of small sample size. A two-sided *P* value < 0.05 was considered significant for all statistical analyses. This study is registered in ClinicalTrials.gov (NCT02749136).

## Results

### Patient characteristics

**Fig. 1:**
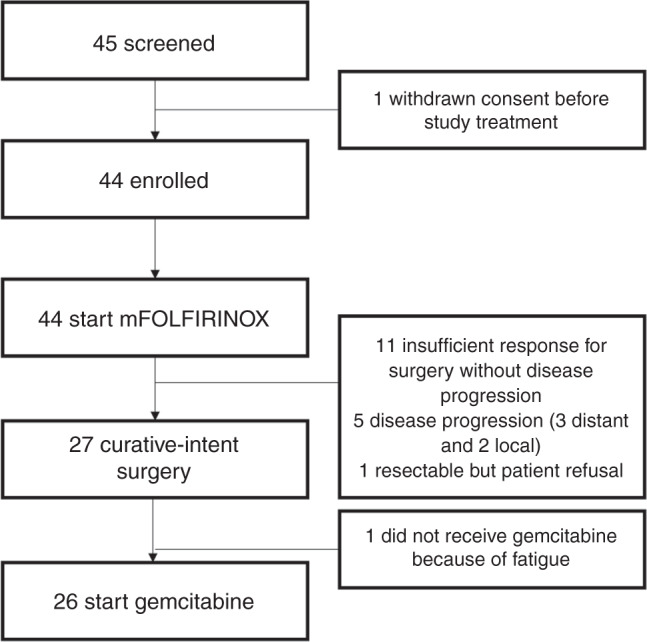
Study flow.

Among the 45 patients who were screened for this study between May 2016 and March 2018, a total of 44 patients were enrolled and received study treatment (Fig. 1). The patients’ baseline characteristics are summarised in Table 1. The median age was 60 years (range, 35–76), and 59.1% of patients were male. The most common primary tumour site was the head of pancreas (*n* = 26, 59.1%), followed by the body (*n* = 14, 31.9%) and tail (*n* = 2, 4.5%). Major vein and artery invasion were noted in 20 (45.5%) and 6 (13.6%) patients, respectively, and 18 (40.9%) patients had involvement of both major veins and arteries. According to the blinded central image review, 35 (79.5%) and 9 (20.5%) patients were classified to have BRPC and LAUPC, respectively.

**Table 1:** Patient Characteristics.

	Total (*N* = 44)
Age (years)	
Median (range)	60 (35–76)
<60 years	22 (50.0%)
≥60 years	22 (50.0%)
Sex	
Male	26 (59.1%)
Female	18 (40.9%)
Primary tumour site	
Head	26 (59.1%)
Body	14 (31.9%)
Tail	2 (4.5%)
Multifocal	2 (4.5%)
ECOG Performance status	
0–1	44 (100%)
Baseline serum CA 19-9 level	
Within normal range	11 (25.0%)
>Upper normal limit	33 (75.0%)
Tumour size	
Median (range), cm	3.3 (1.5–6.1)
Vessel involvement	
Venous	20 (45.5%)
Arterial	6 (13.6%)
Both	18 (40.9%)
None	0 (0%)
Disease extent by blinded central review	
Borderline resectable	35 (79.5%)
Locally advanced unresectable	9 (20.5%)

*ECOG* Eastern Cooperative Oncology Group.

Among the 44 patients who received neoadjuvant mFOLFIRINOX, 27 (61.4%) underwent curative-intent surgery (Fig. 1). The most common reasons for forgoing surgery were insufficient tumour response for resection without disease progression (*n* = 11), and progressive disease (*n* = 5, 3 new distant metastasis and two local progression). One patient refused surgery despite conversion to resectable disease after mFOLFIRINOX. Except for 1 patient who experienced postoperative fatigue, 26 (96.3%) patients received postoperative gemcitabine. Among 12 patients who remained unresectable after 8 cycles of mFOLFIRINOX, 3 and 4 patients received radiotherapy before and after progression on mFOLFIRINOX, respectively.

### Efficacy

With neoadjuvant mFOLFIRINOX, partial responses were achieved in 15 (34.1%) patients, and no patients had a complete response, indicating an objective response rate (ORR) of 34.1% (Table 2). Stable disease and progressive disease were the best responses in 28 (63.6%) and 1 (2.3%) patients, respectively.

**Table 2:** Efficacy Outcomes.

Outcome	Total (*N* = 44)
Best response to preoperative mFOLFIRINOX	
Partial response	15 (34.1%)
Stable disease	28 (63.6%)
Progressive disease	1 (2.3%)
1-year PFS rate (95% CI)	52.3% (37.6–67.0)
2-year OS rate (95% CI)	50.8% (34.5–67.1)
Median PFS, months (95% CI)	12.2 (8.9–15.5)
Median OS, months (95% CI)	24.7 (12.6–36.9)
Surgery	27 (61.4%)
Median DFS from surgery, months (95% CI) (*n* = 27)	10.4 (9.2–11.6)

*PFS* progression-free survival, *OS* overall survival, *DFS* disease-free survival.

**Fig. 2:**
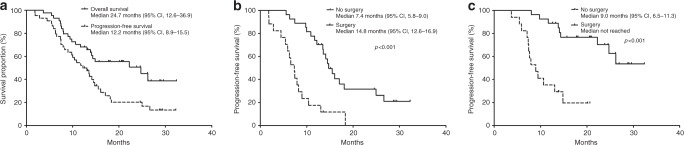
Survival outcomes. PFS and OS for overall patients (**a**), PFS according to the surgery (**b**) and OS according to the surgery (**c**). PFS, progression-free survival; OS, overall survival.

With a median follow-up duration of 20.6 months (95% CI, 19.7–21.6 months), the median PFS and OS were 12.2 months (95% CI, 8.9–15.5 months) and 24.7 months (95% CI, 12.6–36.9), respectively (Fig. 2a). The 1- and 2-year PFS rates were 52.3% (95% CI, 37.6–67.0%) and 20.2% (95% CI, 7.3–33.1%), respectively. The 1- and 2-year OS rates were 68.2% (95% CI, 54.5–81.9%) and 50.8% (34.5–67.1%), respectively.

The details of the surgical findings for the 27 patients who underwent surgery are summarised in Table 3. While there was no patient with R2 resection, 22 (81.5%) patients achieved microscopic complete (R0) resection, and 5 (18.5%) patients had microscopic residual disease (R1 resection). According to the AJCC 8th edition, 24 (88.9%) and 3 (11.1%) patients had T1/T2 or T3 stage, respectively. Lymph node metastasis was negative in 17 (63.0%) patients, and 8 (29.6%) and 2 (7.4%) patients had N1 and N2 stage, respectively. Major vein and artery resection were performed in 11 (40.7%) and 6 (22.2%) patients, respectively. For patients who underwent surgery, median PFS and OS were significantly better than for those who did not (PFS, median 14.8 months [95% CI, 12.6–16.9 months] vs 7.4 months [95% CI, 5.8–9.0 months], *P* < .001; OS, not reached vs 9.0 months [95% CI, 6.5–11.3 months], *P* < .001; Fig. 2b, c).

**Table 3:** Details of surgery and pathological outcomes of resected patients.

Variables	Total (*n* = 27)
Procedure	
Pancreaticoduodenectomy	17 (63.0%)
Subtotal/distal pancreatectomy	9 (33.3%)
Total pancreatectomy	1 (3.7%)
Margin status	
R0 resection	22 (81.5%)
R1 resection	5 (18.5%)
Major vessel resection	
Vein resection	11 (40.7%)
Artery resection	6 (22.2%)
T stage (AJCC 8^th^)	
T1	7 (25.9%)
T2	17 (63.0%)
T3	3 (11.1%)
T4	0
N stage (AJCC 8^th^)	
N0	17 (63.0%)
N1	8 (29.6%)
N2	2 (7.4%)
Tumour differentiation	
Well differentiated	4 (14.8%)
Moderately differentiated	21 (77.8%)
Poorly differentiated	2 (7.4%)

*AJCC* American Joint Committee on Cancer.

The results of the analyses for the association between survival outcomes and clinical characteristics are listed in Table 4. Objective response (CR or PR) to preoperative mFOLFIRINOX was associated with better OS compared with the response of stable disease or progressive disease (median 26.2 months [95% CI, 22.7–29.8 months] vs 13.8 months [95% CI, 9.7–17.9 months], *P* = 0.046), although the relationship between response and PFS was not statistically significant (median 14.8 months [95% CI, 12.5–17.0 months] vs 9.8 months [95% CI, 6.7–12.9 months], *P* = 0.09; Supplementary Fig. 1A, B). Primary tumour site was also significantly associated with survival outcomes (head vs body/tail; PFS, median 14.1 months [95% CI, 11.3–17.0 months] vs 7.5 months [95% CI, 4.9–10.0 months], *P* = 0.02; and OS, median 26.2 months [95% CI, not assessable] vs 11.7 months [95% CI, 5.9–17.4 months], *P* = 0.02; Supplementary Fig. 1C, D). Sex, age (<60 years vs ≥60 years) and baseline CA 19-9 level (within vs >upper normal range) were not associated with either PFS or OS (all for *P* > 0.05).

**Table 4:** Analyses for the association between survival outcomes and clinical characteristics.

Variables	Progression-free survival		
	Unadjusted hazard ratio	95% Confidence interval	*P* value
Sex (female vs male)	1.34	0.67–2.68	0.41
Age (≥ vs <60 years)	0.80	0.41–1.57	0.52
Baseline serum CA 19-9 levels (elevated vs within normal range)	1.18	0.68–1.18	0.68
Primary tumour site (head vs body/tail)	0.44	0.22–0.90	0.02
Response to mFOLFIRINOX (CR/PR vs SD/PD)	0.54	0.26–1.12	0.09
Disease extent by central review (LAUPC vs BRPC)	1.00	0.41–2.43	1.00
Conversion surgery (yes vs no)	0.23	0.11–0.47	<0.001
*Variables*	*Overall survival*		
Sex (female vs male)	1.52	0.65–3.55	0.34
Age (≥ vs <60 years)	0.67	0.28–1.60	0.37
Baseline serum CA 19-9 levels (elevated vs within normal range)	0.68	0.28–1.67	0.40
Primary tumour site (head vs body/tail)	0.36	0.15–0.85	0.02
Response to mFOLFIRINOX (CR/PR vs SD/PD)	0.37	0.14–1.02	0.046
Disease extent by central review (LAUPC vs BRPC)	1.25	0.46–3.39	0.66
Conversion surgery (yes vs no)	0.14	0.05–0.39	<0.001

*CR* complete response, *PR* partial response, *SD* stable disease, *PD* progressive disease, *LAUPC* locally advanced unresectable pancreatic cancer, *BRPC* borderline resectable pancreatic cancer.

Between patients with BRPC and LAUPC redefined by the blinded central image review, there were no statistical differences in terms of conversion surgery rates (62.9% [22/35] vs 55.6% [5/9]; *P* = 0.72), ORR (31.4% vs 44.4%, *P* = 0.76), PFS (median 13.0 months [95% CI, 9.0–17.1 months] vs 12.2 months [95% CI, 10.7–13.8 months]; *P* = 1.00), and OS (median 22.2 months [95% CI, not assessable] vs 24.7 months [95% CI, 2.4–47.1 months]; *P* = 0.66) (Supplementary Fig. 1E, F).

After surgery, 19 (70.4%) of 27 patients experienced recurrence, with a median disease-free survival (DFS) from surgery of 10.4 months (95% CI, 9.2–11.6 months). Median OS from surgery was not reached, and the OS rate at 2 years after surgery was 54.4% (Table 2 and Supplementary Fig. 2). Pattern of recurrences were as follows: 11 (57.9%) at distant sites, 8 (42.1%) at local sites, and 3 (15.8%) at both local and distant sites. The liver was the most common recurrence site (*n* = 9, 47.4%), followed by peritoneum (*n* = 5, 26.3%), lymph nodes (*n* = 3, 15.8%) and lung (*n* = 3, 15.8%). Median DFS from surgery was significantly associated with resection margin status (R0, 10.7 months [95% CI, 6.7–14.8 months] vs R1, 4.0 months [95% CI, 3.4–4.6 months]; *p* = 0.004) and T stage (T1/T2, 10.7 months [95% CI, 8.1–13.3 months] vs T3, 4.0 months [95% CI, 0.0–8.8 months]; *p* < 0.001), while tumour differentiation and lymph node metastasis were not associated with median DFS from surgery (*p* = 0.58 and *p* = 0.38, respectively).

### Immune cell biomarker analysis

**Fig. 3:**
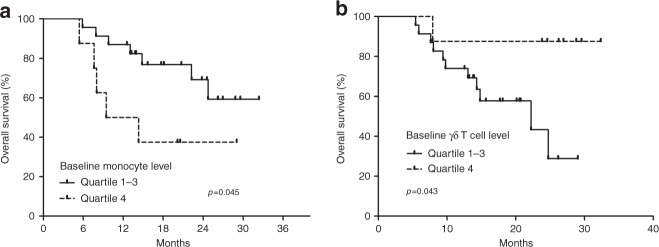
Correlation between overall survival and plasma immune cell subsets. Baseline monocyte level (**a**), baseline γδ T cell level (**b**).

Flow cytometry analysis of PBMC was available for 31 (70.5%) patients. In the correlation analysis between baseline immune cell subsets and OS, higher CD14^+^ monocyte (quartile 4 vs quartiles 1–3) and lower CD69^+^ γδ T cell (γδ TCR^+^/CD69^+^) levels (quartiles 1–3 vs quartile 4) were significantly associated with poor OS (*P* = .045 and *P* = 0.043, respectively) (Fig. 3).

### Safety

Doses of mFOLFIRINOX were interrupted and reduced for 29 (65.9%) and 30 (68.2%) patients, respectively. Three (6.8%) patients discontinued mFOLFIRINOX within 8 cycles due to AEs. The most common grade 3–4 adverse events with mFOLFIRINOX were neutropenia (*n* = 24, 54.5%), anaemia (6, 13.6%), and nausea (5, 11.4%) (Supplementary Table 1). G-CSF was administered to 20 patients (45.5%) during mFOLFIRINOX treatment. Relative dose intensities of oxaliplatin, irinotecan, and 5-FU were at least 75% during mFOLFIRINOX (Supplementary Table 2). In patients who received postoperative gemcitabine (*n* = 26), a median of 5 cycles (range, 1–6 cycles) were administered, and doses of gemcitabine were interrupted and reduced for 10 (38.5%) and 16 (61.5%) patients, respectively. The most common grade 3–4 AEs with postoperative gemcitabine were neutropenia (*n* = 14, 53.8%) and thrombocytopenia (*n* = 3, 11.5%). The relative dose intensity of gemcitabine was maintained at least 78% within 3 cycles (Supplementary Table 3).

## Discussion

In this study, perioperative chemotherapy consisting of 8 cycles of neoadjuvant mFOLFIRINOX and 6 cycles of postoperative gemcitabine was feasible for BRPC/LAUPC patients. The rate of conversion surgery was 61.4%, and the 1-year PFS rate was 52.3%, which indicates that this study met its primary endpoint cut-off (>50%). The median PFS and OS were 12.2 months and 24.7 months, respectively. Although this study was intended to include only patients with BRPC, and documentation of borderline resectability on multiphasic CT and MRI scans was mandatory, the blinded central image review revealed that 20.5% of our study population had LAUPC at baseline. This may be explained by inter-observer variability among radiologists on the determination of BRPC.^11^

Our efficacy outcomes for neoadjuvant mFOLFIRINOX are in line with the results of previous prospective trials, retrospective studies, and meta-analyses. In 2 prior single-arm Phase 2 studies using 8 cycles of neoadjuvant FOLFIRINOX and following radiotherapy for patients with BRPC and LAUPC, surgical resection rates were 67%-69%, and median PFS and OS were 14.7–17.5 months and 31.4–37.7 months, respectively.^12,13^ A series of small retrospective analyses for FOLFIRINOX-based neoadjuvant therapy for BRPC and LAUPC showed 40–80% of resection rates.^6^ In a recent patient-level meta-analysis for neoadjuvant FOLFIRINOX, including 283 BRPC patients from 20 studies, the surgical resection rate was 67.8% (R0 rate 83.9%), and the median PFS and OS were 18.0 months and 22.2 months, respectively.^14^

In this study, patients received gemcitabine monotherapy after surgery, because this study was designed and conducted before the data of ESPAC-04 and PRODIGE trials, which demonstrated a survival benefit with postoperative gemcitabine plus capecitabine and mFOLFIRINOX as postoperative chemotherapy for resected pancreatic cancer.^3,4^ In 27 patients who underwent surgery after mFOLFIRINOX, 70.4% of patients experiences recurrence, and the median disease-free survival after surgery was 10.4 months. Considering the high recurrence rates even among patients who underwent curative-intent surgery, more effective adjuvant chemotherapy regimens, such as gemcitabine plus capecitabine or mFOLFIRINOX should be used for medically fit patients following the neoadjuvant mFOLFIRINOX and surgery. The optimal duration and regimens of postoperative chemotherapy for patients who undergo conversion surgery after neoadjuvant mFOLFIRINOX should be defined in future studies.

As prognostic factors, surgical resection and better response to neoadjuvant mFOLFIRINOX were significantly associated with better OS. Among baseline factors, sex, age and baseline CA 19-9 levels were not associated with clinical outcomes in this study. Interestingly, there was no difference in terms of PFS and OS between BRPC and LAUPC in our study. This in in line with the results of a recent patient-level meta-analysis,^14^ which found no difference in median OS between BRPC and LAUPC.

Despite recent advances in neoadjuvant therapy using mFOLFIRINOX in BRPC, further improvements in its efficacy are needed considering that only two-thirds of patients undergo surgery and more than half of patients experience recurrence even after surgery less than 2 years postoperatively. In our study, the most common reason for forgoing surgery (11 of 17 patients) was insufficient tumour response for surgery without tumour progression, and 42.1% of patients whose disease relapsed after curative-intent surgery had local recurrence only. These data suggest the potential benefits of additional preoperative or postoperative radiotherapy to mFOLFIRINOX-based neoadjuvant therapy for BRPC and LAUPC. This approach may improve the local tumour response and surgical resection rates associated with better survival outcomes. A randomised Phase 2 trial (ALLIANCE A021501 trial) comparing 8 cycles of mFOLFIRINOX and 7 cycles of mFOLFIRINOX followed by hypo-fractionated radiotherapy is ongoing for BRPC of the head of pancreas, and this will reveal the implications of radiotherapy in the era of modern chemotherapy for localised pancreatic cancer.^15^

In our biomarker analysis using immune cell subsets from baseline PBMC samples, high monocyte and low γδ T cell levels were significantly associated with worse OS. The negative prognostic implication of monocytes has been reported in multiple cancer types including pancreatic cancer,^16^ and the promising efficacy of CCR2 blockade targeting monocytes in combination with FOLFIRINOX suggests the potential role of monocytes in pancreatic cancer.^17^ γδ T cells are MHC-unrestricted unconventional lymphocytes and known to have protective roles against cancer based on their potent cytotoxicity and interferon-γ production.^18^ Our results suggest that γδ T cells might play some role in pancreatic cancer; however, γδ T cells as therapeutic targets remains to be further studied, and there is a lack of in vivo proof-of-concept human data.

This study has certain limitations. The study was performed at the single centre and about 20% of patients had LAUPC, despite this study was originally intended to investigate only BRPC. The number of included patients was also too small to validate the predictive marker in the multivariate analysis.

In conclusion, neoadjuvant mFOLFIRINOX followed by postoperative gemcitabine were effective for BRPC patients. Biomarker analyses revealed the potential role of immune cell subsets in pancreatic cancer.

## Supplementary information

Supplementary Materials

## Data Availability

The data that support the findings of this study are available from the corresponding author upon reasonable request.
