# Supplementary material for: Neoadjuvant modified FOLFIRINOX followed by postoperative gemcitabine in borderline resectable pancreatic adenocarcinoma: a Phase 2 study for clinical and biomarker analysis
Source: Br J Cancer. 2020 May 20;123(3):362–8. doi: 10.1038/s41416-020-0867-x (PMC7403346; doi:10.1038/s41416-020-0867-x)

**Supplementary Table 1. Safety Profiles with Neoadjuvant mFOLFIRINOX and Postoperative Gemcitabine**

| Adverse Events | Neoadjuvant mFOLFIRINOX (n=44) | | Postoperative Gemcitabine (n=26) | |
| --- | --- | --- | --- | --- |
|  | All grades | Grade 3-4 | All grades | Grade 3-4 |
| Neutropenia | 31 (70.5%) | 24 (54.5%) | 18 (69.2%) | 14 (53.8%) |
| Febrile neutropenia | 3 (6.8%) | 3 (6.8%) | 0 | 0 |
| Anemia | 24 (54.5%) | 6 (13.6%) | 15 (57.7%) | 2 (7.7%) |
| Thrombocytopenia | 5 (11.4%) | 3 (6.8%) | 14 (53.8%) | 3 (11.5%) |
| Anorexia | 17 (38.6%) | 2 (4.5%) | 11 (42.3%) | 0 |
| Nausea | 25 (56.8%) | 5 (11.4%) | 10 (38.5%) | 1 (3.8%) |
| Vomiting | 12 (27.3%) | 1 (2.3%) | 3 (11.5%) | 1 (3.8%) |
| Abdominal pain | 16 (36.4%) | 2 (4.5%) | 13 (50.0%) | 0 |
| Alopecia | 6 (13.6%) | 0 | 1 (3.8%) | 0 |
| Diarrhea | 19 (43.2%) | 3 (6.8%) | 9 (34.6%) | 0 |
| Constipation | 3 (6.8%) | 0 | 3 (11.5%) | 0 |
| Fatigue | 14 (31.8%) | 0 | 8 (30.8%) | 1 (3.8%) |
| Myalgia | 7 (15.9%) | 0 | 6 (23.1%) | 0 |
| Peripheral neuropathy | 10 (22.7%) | 0 | 10 (38.5%) | 0 |
| Stomatitis | 7 (15.9%) | 1 (2.3%) | 3 (11.5%) | 0 |

**Supplementary Table 2. Relative Dose Intensity of mFOLFIRINOX**

|  | Cycle 1 | Cycle 2 | Cycle 3 | Cycle 4 | Cycle 5 | Cycle 6 | Cycle 7 |
| --- | --- | --- | --- | --- | --- | --- | --- |
| **No. of patients** | **44** | **43** | **43** | **40** | **38** | **37** | **35** |
|  | **Mean (SD)** | **Mean (SD)** | **Mean (SD)** | **Mean (SD)** | **Mean (SD)** | **Mean (SD)** | **Mean (SD)** |
| **Oxaliplatin** | 79.8% (19.4) | 88.9% (16.7) | 91.1% (19.0) | 87.1% (19.6) | 81.8% (20.3) | 86.8% (19.1) | 79.1% (17.0) |
| **Irinotecan** | 76.7% (19.9) | 83.4% (19.0) | 84.8% (20.5) | 82.5% (21.9) | 76.8% (22.4) | 81.6% (21.6) | 77.9% (18.6) |
| **5-FU** | 80.1% (19.2) | 89.0% (16.6) | 91.4% (18.6) | 87.7% (19.5) | 82.6% (19.5) | 87.8% (18.2) | 80.3% (16.4) |

Abbreviation: SD, standard deviation

**Supplementary Table 3 Relative Dose Intensity of Postoperative Gemcitabine**

|  | Cycle 1 | Cycle 2 | Cycle 3 | Cycle 4 | Cycle 5 | Cycle 6 |
| --- | --- | --- | --- | --- | --- | --- |
| **No. of patients** | 26 | 24 | 21 | 15 | 14 | 13 |
|  | Mean (SD) | Mean (SD) | Mean (SD) | Mean (SD) | Mean (SD) | Mean (SD) |
| **Gemcitabine** | 83.4% (21.5) | 87.0% (14.5) | 77.9% (21.0) | 67.8% (22.2) | 73.2% (15.2) | 73.4% (16.3) |

Abbreviation: SD, standard deviation

**Supplementary Figure 1. Subgroup analysis**

Response to mFOLFIRINOX (A for PFS, B for OS), primary tumor location (C for PFS, D for OS), and disease extent (E for PFS, F for OS).

PFS=progression-free survival, OS=overall survival

**
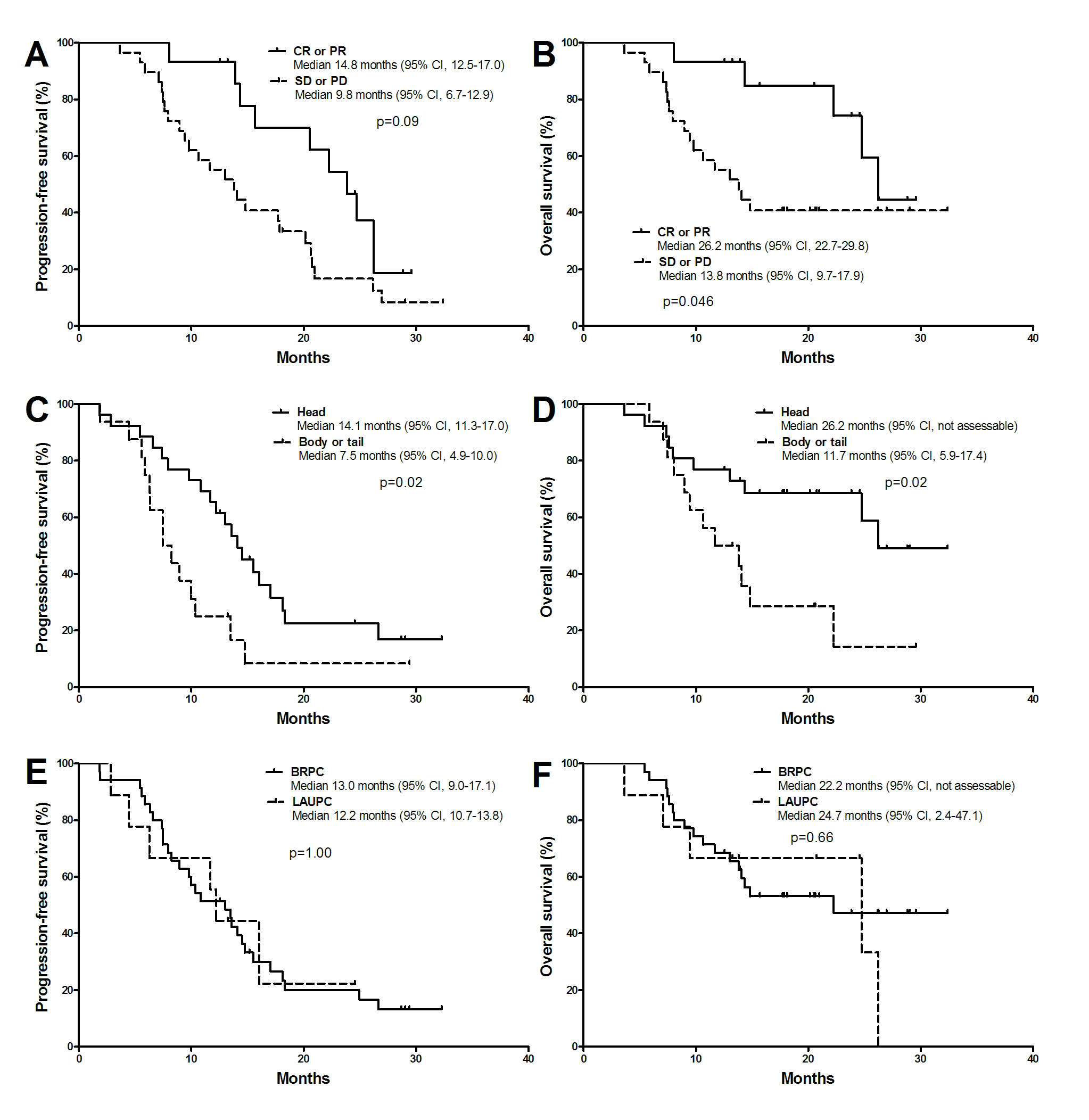
**

**Supplementary Figure 2. Disease-free Survival and Overall Survival Since Surgery**


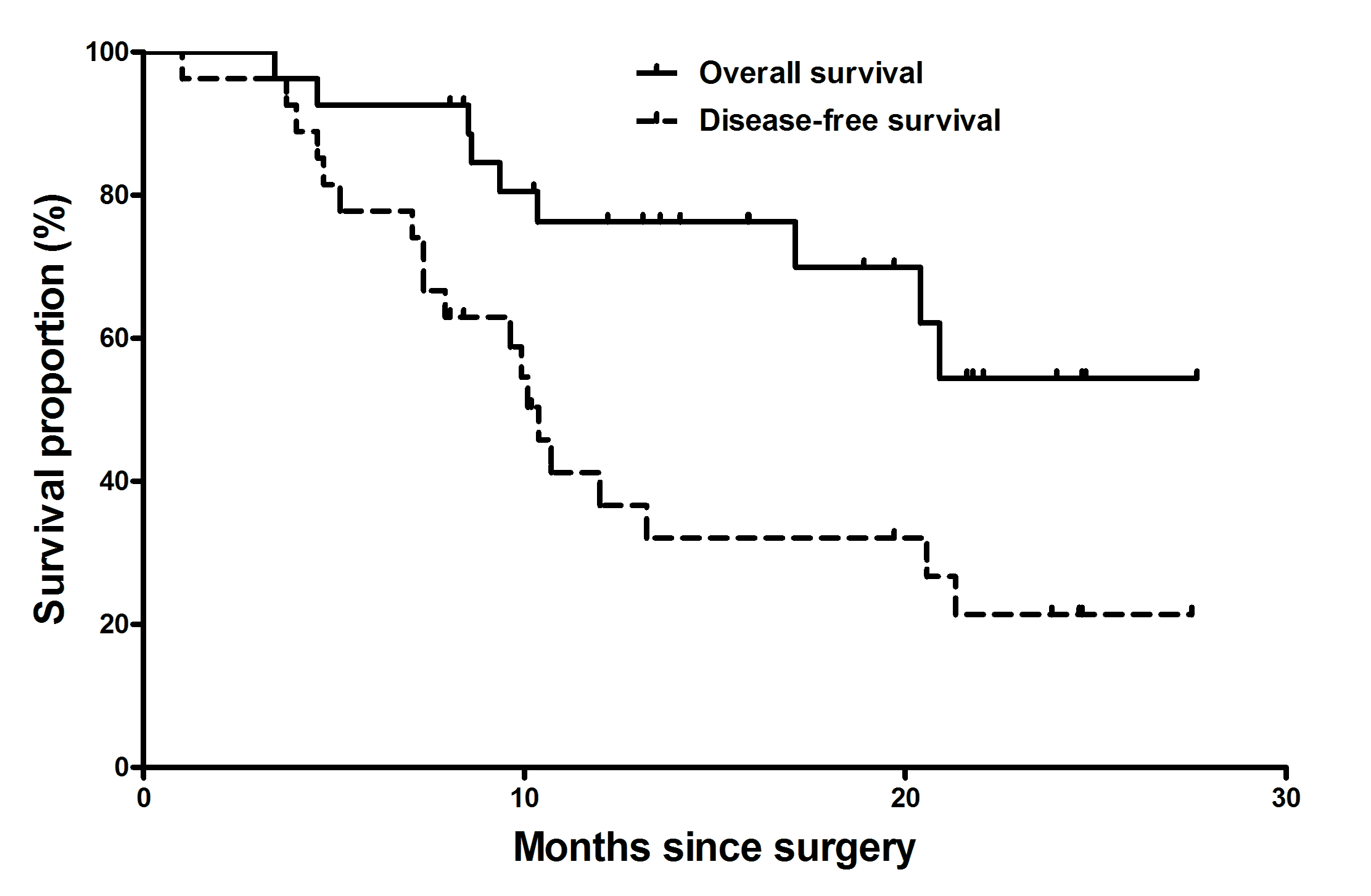

Supplement: Supplementary file 1 — Supplementary Materials [file 41416_2020_867_MOESM1_ESM.docx]
